# Supporting Emergency Department Patients Experiencing Homelessness

**DOI:** 10.1016/j.acepjo.2025.100310

**Published:** 2025-12-29

**Authors:** Antony Hsu, Michael A. Light, Callan Fockele, Samantha Hay, Stephen Y. Liang, Wendy Macias-Konstantopoulos, Carla B. Brim, William Weber

**Affiliations:** 1 Department of Emergency Medicine, Trinity Health Ann Arbor, Ypsilanti, Michigan, USA; 2 Harborview Medical Center, University of Washington, Seattle, Washington, USA; 3 Downtown Emergency Services Center, University of Washington, Seattle, Washington, USA; 4 Department of Emergency Medicine, University of Illinois in Chicago, Chicago, Illinois, USA; 5 Department of Emergency Medicine and Division of Infectious Diseases, Department of Medicine, Washington University School of Medicine, St. Louis, Missouri, USA; 6 Department of Emergency Medicine, Massachusetts General Hospital, Boston, Massachusetts, USA; 7 Emergency Nurse Association, Schaumberg, Illinois, USA; 8 Department of Emergency Medicine, Rush University Medical Center, Chicago, Illinois, USA

## Abstract

Homelessness is a growing public health crisis across the United States. Emergency physicians are uniquely positioned to address immediate medical concerns and the underlying social drivers of health for patients experiencing homelessness. Initiating coordination of care in the emergency department addresses their hierarchy of needs and can help support patient movement through the department. Practice adaptations described include early engagement; trauma-informed care approach; addressing unmet nonmedical needs; establishing safe dispositions; documenting homelessness, substance use disorder, and linkages to care; clinical practice adaptations; and palliative care approaches. Programs and initiatives outside of the emergency department described include street medicine and government initiatives. Sustainable solutions offered ideally use programs and incentives already available.

## 1 Background

Homelessness is a growing public health crisis across the United States with over 650,000 individuals sleeping unhoused in 2023, representing a 12% increase from the previous year and accelerated to 770,000 individuals in 2024, an 18% increase from 2023.^1^^,^^2^ While homelessness is a transitory state for many people, for the approximately 31% experiencing chronic homelessness, lack of stable housing can limit access to health care and the ability to adhere to medical treatment.^3^ These factors lead to higher rates of preventable and treatable illness and a mortality rate of 4 to 9 times higher than their housed peers, with an average life expectancy of just 48 to 52 years.^3^^,^^4^ The emergency department (ED) is sometimes the most easily accessible point of health care contact for unhoused community members, leaving emergency physicians (EPs) uniquely positioned to address immediate medical concerns and the underlying social drivers of health. This article integrates current literature and lessons from innovative health care programs to provide insight on the multiple needs of patients experiencing homelessness and practice adaptations within the ED for bedside evaluation and management.

This article favors the term patients experiencing homelessness (PEH), as it minimizes the negative stigma and inaccuracies associated with the terms homeless, houseless, and unhoused. According to the Health Resources and Services Administration (HRSA), PEH encompasses anyone who lacks housing including those sleeping in supervised temporary living accommodations (eg, shelters) and those who reside in transitional housing or permanent supportive housing.^5^ Data from US Department of Housing and Urban Development estimates that approximately 60% of PEH are living sheltered, leaving 40% living unsheltered on the street or in encampments.^2^

National data have reported downtrends in homelessness for youths, families, and veterans in the past decade.^6^ However, intersectional disparities in homelessness persist with numbers climbing significantly for some marginalized groups including transgender people, Asians, Latinos, American Indians, and newly arrived immigrants. An estimated 1 in 4 PEH are 55 years and older, which is expected to triple by 2030.^2^^,^^7^ Given that PEH experience geriatric conditions such as memory loss, falls, and functional impairments at a greater rate and at a younger age, PEH over the age of 50 years are often designated as older adults.^8^^,^^9^ Often framed as an urban issue, approximately 4 of 10 individuals and families live unhoused in rural and suburban areas, are often undercounted and unrecognized, and may have distinct service and policy needs.^5^

ED visit rates for PEH doubled from 2011 to 2021, while visit rates for housed peers remained stable over the same period.^10^ PEH seek ED care for many reasons, including scant access to community-based health and human service resources, limited hours at primary care clinics, and lack of transportation.^11^ The ED provides access to safe, climate-controlled spaces that are open 24/7, which can be reached via emergency medical services if patients lack funds or other means of travel. PEH can be perceived to overuse the ED, but a national sample that adjusted for medical, mental health, and social factors found similar rates of use compared with those by housed peers.^12^ While EDs can provide care for acute medical needs and access to routine medications and medical supplies, the current acute care–centered, episodic model of emergency medicine is not designed address the root causes of frequent ED visits and hospital admissions by PEH.^13^ PEH have higher rates of admission than housed peers for substance use disorder and mental illness, suggesting that better outpatient substance use and psychiatric resources could potentially help reduce inpatient reliance.^14^ Bolstering investment in case management services to support complex discharge planning and resource linkage are worthy options for research to appropriately mitigate the visit frequency for PEH.^15^ An integrated discharge and referral system between primary care, housing, and social care that addresses the needs of PEH may reduce the need for ED visits. Such a system can ultimately improve health and social care coordination, outpatient health care maintenance, and health outcomes.^16^ This article describes potential practice adaptations for EPs caring for PEH that can help support their needs, both during the ED visit and after discharge.

## 2 ED Interactions with PEH

EPs can maximize the positive impact of ED encounters by addressing acute medical and social needs and linking patients to community resources.^17^ Patients, including those with significant social needs, often require multiple hours in the ED for evaluation and treatment. This time can provide particularly effective opportunities for case managers, patient navigators, social workers, and other health and social service coordinators to understand the priorities and care goals of PEH, enroll patients in hospital-based services, or set up direct referrals to community services. Although the literature is mixed on whether intensive care coordination programs effectively reduce ED utilization, an integrated discharge and referral between primary care, housing services, and social care provides an opportunity to mitigate social risk, link PEH to community resources, and coordinate outpatient health care services.^18^, ^19^, ^20^ Figure diagrams the coordination of care in the ED that addresses the hierarchy of needs of PEH.

**Figure:**
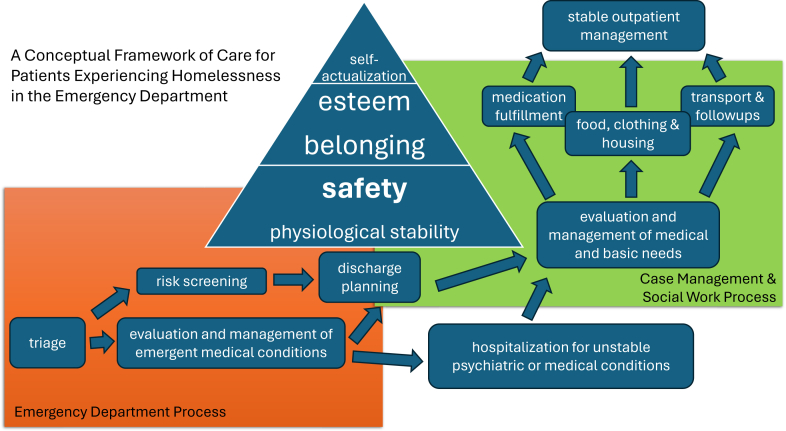
Emergency department (ED) coordination of care for patients experiencing homelessness (PEH).

Not all EDs where PEH seek care have access to case managers or social workers. Innovative methods of engagement are being developed, including conversational agents chatbots via handheld devices or kiosks.^21^^,^^22^ Although these chatbots are less helpful for highly health-literate and well-resourced patients, PEH with low health literacy may benefit from social services education or health screenings while in the ED.^23^^,^^24^ Further medical care for the truly unhoused may come in the form of street medicine programs which are further described in Tables S4 and S5.

## 3 Practice Adaptations within the ED for PEH

### 3.1 Early Engagement

Rapid recognition of social needs by EPs can lead to incremental and deliberate steps to address these needs. Determining the main objectives for the visit (eg, access to food and daily medications) can help guide engagement of social service coordinators during an ED visit. Early involvement of social services allows more time to coordinate hospital and community resources for PEH and lowers the risk of a delayed disposition.

- Initiate referrals to services early in the visit to reduce delays in disposition for social reasons.

### 3.2 Trauma-Informed Care Approach

Trauma-informed care refers to a strengths-based framework that seeks to inform services about the impact of trauma and minimize retraumatization of individuals accessing care that has influenced the provision of behavioral healthcare for the past decade.^25^^,^^26^ EPs, case managers, and multidisciplinary teams can engage PEH using a trauma-informed care approach to determine reasons for the ED visit and respond to health-related social needs and adverse experiences with empathy.^25^^,^^27^ EPs should be mindful of how dismissive body language or vocabulary in their interactions can contribute to stigmatization of these vulnerable patients and lead to decreased patient trust. Acknowledging each patient’s humanity and their rationality in seeking support (even if the ED is not the best use of resources) can help foster a therapeutic relationship.

- Adopt a trauma-informed approach to care for PEH.
- Avoid stigmatizing language and actions.

### 3.3 Addressing Unmet Social Needs in the ED

Many PEH presenting to the ED for care may be experiencing concurrent social emergencies, including a lack of basic needs, such as food and warm clothing. Most obvious is the need for stable housing. For many patients, homeless shelters or other temporary housing may be the only option and are discussed further. Some organizations have focused on a “housing first” model to prioritize placement in a stable living environment before addressing other issues such as substance use. A housing first policy is associated with decreased ED use and fewer hospitalizations, although health outcomes are similar.^27^

EDs can consider programs to offer other services directly onsite. Local needs can guide creative solutions to support basic unmet needs of PEH. Some EDs have developed integrated food pantries, clothes closets, and pharmacies to help PEH achieve medication compliance.^28^^,^^29^ Hospitals with inpatient or outpatient pharmacies willing to fill and package prescriptions can have them delivered to ED patients. One institution with a high rate of poverty, mental health, and substance use implemented “to go” prescriptions at ED discharge and found much lower rates of return visits and admissions, with a program cost of $4050 leading to an institutional cost savings of $95,326.^30^ At an author institution, the ED repurposed a storage closet into a clothes closet for patients who needed clothes or footwear. The closet was stocked by medical staff–led clothing drives and maintained by volunteers. Patients who lacked basic clothes could receive temporary articles of clothing, which facilitated their disposition. These types of stopgap measures can assist PEH while they arrange more permanent supportive services.

- Adopt mechanisms to deliver prescriptions directly to patients at discharge.
- Implement ED-based systems to address patient needs such as clothing.

### 3.4 Establishing Safe Dispositions

EPs should work with hospital leadership to establish financially sustainable and equity-minded protocols to support safe disposition of PEH at all times of the day. This coordination can help support patient movement through the department as EDs often struggle with disposition of patients who lack shelter or transportation. Close communication with accepting shelters regarding intake criteria preserves working relationships. While mandatory safe shelter and medication access has been legislated at the state level, funding for these mandates is lacking.^31^ Depending on transportation options in the community, hospital-based EDs may provide public transportation passes or, when necessary, cover rideshare or taxi services. One large urban medical center provides direct referrals to hospital-based housing navigators with access to their state or local Homeless Management Information Systems in order to access supportive housing.^32^^,^^33^ For communities where shelters have limited intake hours, having a designated area for discharged patients waiting for the shelter to open can potentially alleviate boarding issues.

- Develop partnerships with local shelters that can receive patients at discharge, ideally with expanded intake hours.
- Evaluate transportation options for PEH to get to their destination.

### 3.5 Documenting Homelessness

EPs play an important role in assessing and documenting social determinants of health (SDOH) including housing security. Much of the data referenced in this paper rely on SDOH documentation in medical records. The current International Classification of Diseases (ICD)-10-CM code for homelessness (Z59.0) is not typically billable as a primary diagnosis in the ED, but its inclusion can add to the complexity and level of service of an encounter.^34^^,^^35^ Identifying PEH with a Z-code is a fast and simple way EPs can provide data to improve interprofessional care coordination, enhance quality improvement initiatives, bring attention and resources to EDs, and monitor SDOH intervention effectiveness. As value-based payment models expand, increased reimbursement can benefit hospitals disproportionately serving PEH. Z-codes may impact the length-of-stay index needed for quality metrics and avoidance of potential audit-based financial penalties.^36^

- Document housing insecurity to improve epidemiology, linkages to care, and capture the extra work required to care for the population of PEH.

### 3.6 Substance Use Disorders and Other Mental Health Disorders and Linkages to Care

Mental health resources remain challenging to obtain in many communities and mental health has a strong and bidirectional effect on housing status, with unstable housing being associated with worse medication compliance and psychiatric decompensation, which makes it difficult to maintain stable housing.^37^ PEH have much higher rates of mental illness than the general population, with approximately two-thirds experiencing mental illness, not only substance use disorders but also significant levels of depression, schizophrenia, and personality disorders.^38^ The stresses of unstable housing can also contribute to worsening symptoms and increased social impairment.

PEH are disproportionately affected by substance use disorder, with overdose being the leading cause of death among PEH in many cities.^39^ The association between substance use and homelessness is complex. PEH may use drugs for adaptive reasons, including staying awake in hazardous environments, addressing untreated trauma and mental illness, and combating the adverse side effects of other substances.^40^ The harms of injection drug use are exacerbated by the challenging environment and conditions experienced during homelessness, which can lead to outdoor injections and lack of access to skin cleansing and hand hygiene facilities.^41^

Many PEH desire outpatient treatment but face significant barriers to traditional addiction management, including limited walk-in hours and strict late arrival policies, which fail to accommodate social complexity.^42^ Inpatient and detox facilities may require PEH to present without their belongings or their animal companions. Pharmacies and opioid treatment programs frequently require patients on controlled substances to show identification, which is difficult to obtain and retain while experiencing homelessness, and prescriptions can be difficult to maintain without consistent access to safe storage. Table 1^43^, ^44^, ^45^, ^46^, ^47^, ^48^, ^49^, ^50^ describes practice adaptations for EPs supporting PEH with various forms of substance use disorder.

**Table 1:** Practice adaptations for PEH who use controlled substances.

Harm reduction	• Provide safer use supplies for injecting (eg, alcohol swabs and clean needles) or allowing needle exchange to lower HIV transmission risk if allowed in your jurisdiction.^43^ • Consider screening for potential sequelae of injection drug use including HIV, hepatitis C, and syphilis and offering pre-exposure prophylaxis (PrEP).
Opioid use disorder	• Provide naloxone prescriptions to patients at risk of opioid overdose. • Offer initiating buprenorphine for patients in opioid withdrawal who are motivated to initiate treatment.^44^^,^^45^
AUD	• Be mindful about initiating long-acting medications for withdrawal such as phenobarbital or chlordiazepoxide in the ED that could potentially compromise the alertness of a PEH who is staying in an unsafe location. Some alternatives such as gabapentin or carbamazepine may be effective and less sedating.^46^^,^^47^ • Prescribe medications such as naltrexone, gabapentin, or acamprosate to reduce cravings and risk of harm from alcohol.^48^ Long-acting injectable naltrexone, particularly when combined with harm reduction treatment, has demonstrated efficacy in decreasing alcohol use and improving the quality of life for PEH.^49^ • Screening, brief intervention, and referral to treatment (SBIRT) can reduce alcohol consumption and risky alcohol-related behavior in ED patients with AUD.^50^
Stimulant use disorder	• Prescribe medications to combat the adverse side effects of stimulant use (eg, melatonin for insomnia).

AUD, alcohol use disorder; PEH, patients experiencing homelessness.

### 3.7 Palliative Care

Palliative care focuses on relieving the symptoms and stress of serious illness while helping to align care to patients’ goals. Palliative care also aligns with harm reduction principles by focusing on quality of life, reducing suffering, elevating patient autonomy, and providing goal-concordant care.^51^ As with other primary and specialty health services, PEH often have limited access to palliative care due to complex comorbidities and psychosocial needs leading to higher rates of early mortality.^4^^,^^52^ As the initial point of care for critical medical events, ED teams are often faced with complex medical decision-making needs for PEH without documented goals of care or advanced care plans. Repeat visits provide EPs with multiple opportunities to address palliative care needs; however, the brevity of ED encounters often limits the extent to which planning can be performed.^53^ Early palliative care interventions should be considered for PEH given their increased risk of morbidity, mortality, and traumatic injury and practice strategies for the ED and a novel program model are described in Table S3.

• Clarify legal surrogate decision makers and identify emergency contacts.
• Ask and document patients’ values, priorities, and preferences related to care over specific decisions for medical care and potential limits for advanced interventions.

### 3.8 Discharge Practice Adaptations

Homelessness is often associated with food insecurity; limited access to clean running water and respite; and exposure to violence, trauma, and communicable diseases, leading to acute conditions like skin and soft tissue infections, exacerbating chronic illnesses like heart failure, and leaving patients with limited resources to address their complex medical needs. Ideal care requires a realistic assessment of systemic barriers and circumstances, with a focus on harm reduction and support of each patient’s goals and priorities. This approach centers on the patient’s expertise in decision making and considers the appropriateness, availability, accessibility, affordability, and acceptability of each medical intervention. Individual care plans should weigh the patient’s comorbidities, side effect burden, medical literacy, transportation access, insurance coverage, and supplies (eg, crusher, splitter, or syringes). Table 2 describes opportunities to adjust practice to reach achievable goals.

**Table 2:** Discharge practice adaptations to lower barriers to community transition.

Medication access	• Discharge PEH with medications in-hand • Use long-acting or single dose antibiotics • Choose longer-lasting medications (eg, torsemide over furosemide, dexamethasone for asthma exacerbations, and injectable PrEP) • Prescribe temperature stable medications • Provide naloxone and sterile injection supplies • Provide syringes or cups to measure liquid medications • Order an inhaler or insulin dose in ED and provide the remainder to patient • Avoid sedating medications unless the patient has a safe place to take them
Reduce follow-up needs	• Use absorbable sutures for lacerations • Provide immunizations if available • Use bivalved casts instead of splints to minimize returns or visits to orthopedic clinics • Provide sufficient dressing or wound-care supplies
Care coordination	• Document social determinants of health for tracking/billing/QI • Consider laboratories for monitoring chronic medications (eg, renal function if on ACE inhibitor) • Provide crutches/splints/boots • Arrange follow-up appointment before discharge • Ask social services staff to help patients apply for Medicaid or similar insurance programs • Document names and office numbers for case managers or other important outpatient referrals • Add chart flags to follow-up on abnormal test results
Nonmedical needs	• Provide sanitary napkins, tampons, and/or condoms • Provide extra socks for keeping feet dry/warm • Provide public transit cards or tokens for transport to shelter and clinic follow-up • Develop hospital policies for safe discharge during temperature advisories • Provide access to bathrooms or shower facilities

ACE, angiotensin-converting enzyme; ED, emergency department; PEH, patients experiencing homelessness; PrEP, pre-exposure prophylaxis.

## 4 Looking Ahead

The causes of homelessness are multifactorial but primarily driven by the lack of affordable housing, the effects of the opioid epidemic, and a limited social safety net. EDs often serve patients at the nexus of medical and social challenges. Structural reforms are desperately needed to address the growing issue of homelessness in the United States beyond individual states addressing supervised injection and current federal government initiatives addressing homelessness (Tables S2 and S6). In the interim, practice adaptations in the ED can help serve a vital role in supporting the needs of PEH.

## Funding and Support

By *JACEP Open* policy, all authors are required to disclose any and all commercial, financial, and other relationships in any way related to the subject of this article as per ICMJE conflict of interest guidelines (see www.icmje.org). The authors have stated that no such relationships exist.

## Conflict of Interest

All authors have affirmed they have no conflicts of interest to declare.
