# Supplementary material for: Supporting Emergency Department Patients Experiencing Homelessness
Source: J Am Coll Emerg Physicians Open. 2025 Dec 29;7(1):100310. doi: 10.1016/j.acepjo.2025.100310 (PMC12803902; doi:10.1016/j.acepjo.2025.100310)
Supplement: Supplementary Tables 1-6 [file mmc1.docx]

Table S1: Why Housing First?

| Housing First programs are based on the philosophy that housing placement should not be contingent on the use of available services or engaging in treatment for substance use and/or mental disorders.^67^ High-quality research consistently shows that Housing First effectively resolves homelessness for most people, with high housing retention rates. While research is mixed on the impact of Housing First on substance use or mental health, there is evidence that permanent supportive housing improves health outcomes among individuals with HIV/AIDS.^68^ |
| --- |

Table S2: Supervised Injection Site Initiatives

| In 2021, New York City opened the first publicly recognized safe injection site while Rhode Island authorized safe injection sites statewide.^69,70^ Several other states, including Colorado, Nevada, and New Mexico, have considered allowing them. On the other hand, the governors of California and Vermont vetoed safe injection site bills in 2023, and Pennsylvania’s Senate voted in 2024 for a ban on safe injection sites.^71–73^ |
| --- |

Table S3: Palliative Care for PEH

| Practice Strategies in the Emergency Department | - Priority should be given to clarifying legal surrogate decision makers and identifying emergency contacts. Partner with psychosocial team members to document contacts and help patients complete legal documents if appropriate. - Document names and contacts for all care team members including community-based professionals like case managers. These team members may offer valuable insight for care planning, discharge planning, and medical decision making. - Build communication skills for goals of care discussions to help guide immediate care and plan for future health crises. Supportive tools include Ariadne Labs’ Serious Illness Conversation Guide and the VitalTalk Tips phone app by VitalTalk.^88,89^ - Use the Patient Dignity Question, a validated tool to initiate serious illness planning conversations by asking, “What do I need to know about you as a person to give you the best care possible?”^90^ - Focus on documenting patients’ values, priorities, and preferences related to care over specific decisions for medical care. Include direct statements about potential limits for advanced interventions. |
| --- | --- |
| Program Model | - Harborview’s Homeless Palliative Care team, based in Seattle, Washington, is the nation’s first mobile outreach program supporting people experiencing homelessness who are living with serious illness. Grounded in a harm-reduction and trauma-informed approach to care, the interprofessional team specializes in relieving the symptoms and stress of serious illness. They partner with medical teams and community-based teams including case managers to improve communication and care coordination to improve health outcomes and align care to patients’ goals.^91^ |

Table S4: Key components of a street medicine program

| Direct medical care | - acute medical care with point-of-care testing and wound care - management of chronic conditions and vaccinations - medication prescribing or distribution - substance use disorder counseling and treatment (e.g., medication-assisted treatment for opioid use disorder); - psychiatric and other mental health services (e.g., crisis screening and intervention) |
| --- | --- |
| Referral pathways to wraparound health services | - locally accessible primary care and subspecialty care - dental care - counseling and other mental health services - substance use disorder treatment programs - medical respite^92^ |
| Harm reduction services | - overdose education and reversal supplies (e.g., intranasal or injectable naloxone for opioid overdose) - safer injection education and supplies (e.g., syringe and needle exchange) - wound care kits; safer sex education and supplies (e.g., condoms) |
| Provision of basic living supplies | - food, water, clothing and hygiene kits - blankets, sleeping bags & tents |
| Case management | - referral to social services and shelter intake or housing programs, if desired - assistance with vital documents (e.g., government-issued identification) - insurance enrollment - transportation for appointments and cell phone support |

Table S5: Best practices that street medicine programs should include:

| Formal organizational structure | - clearly define team member roles and responsibilities - include scope of practice based on training and credentialing |
| --- | --- |
| Standardize treatment protocols and referral procedures | - request periodic reviews by legal counsel and risk management when affiliated with a healthcare institution |
| Safe medication storage and dispensing | - implement procedures to limit access to credentialed prescribers, track utilization, monitor expiration dates, and maintain regulatory compliance - consult pharmacists in developing these processes |
| Robust partnerships | - partner with community-based organizations serving the unsheltered (including street guides with lived experience of homelessness), shelters and other housing authorities - engage law enforcement, public health, mental health and substance use disorder treatment programs |
| Protection of patient privacy | - use secure medical documentation - abide by HIPAA |
| Liability coverage | - review medical malpractice insurance policies - understand state-specific limitations to Good Samaritan laws |

Table S6: Examples of Federal Initiatives addressing homelessness.

| General populations | - HUD has implemented initiatives, such as the Continuum of Care (CoC) Program, which coordinates federal funding for nonprofit providers and state and local governments to avoid duplication of support. - The Emergency Solutions Grants Program (ESG) assists people with acute housing crises by providing street outreach, emergency shelter, homelessness prevention, rapid re-housing assistance and administrative coordination.^93^ - Title V of the McKinney-Vento Homeless Assistance Act, Public Law 101-645 (42 U.S.C 11411) allows organizations to use excess federal properties to assist PEH and should be considered for communities who are initiating programs.^94,95^ |
| --- | --- |
| Specific populations | - HUD supports programs for youth (<25yo), individuals with AIDS, and veterans who identify as PEH.^96–98^ - The Veterans Administration also has housing supports for veterans who identify as PEH.^99^ |
